# Two DNA vaccines protect against severe disease and pathology due to SARS-CoV-2 in Syrian hamsters

**DOI:** 10.1038/s41541-022-00461-5

**Published:** 2022-04-26

**Authors:** George Giorgi Babuadze, Hugues Fausther-Bovendo, Marc-Antoine deLaVega, Brandon Lillie, Maedeh Naghibosadat, Nariman Shahhosseini, Michael A. Joyce, Holly A. Saffran, D. Lorne Tyrrell, Darryl Falzarano, Chandrika Senthilkumaran, Natasha Christie-Holmes, Steven Ahn, Scott D. Gray-Owen, Arinjay Banerjee, Samira Mubareka, Karen Mossman, Chanel Dupont, Jannie Pedersen, Mark-Alexandre Lafrance, Gary P. Kobinger, Robert Kozak

**Affiliations:** 1 Biological Sciences Platform, University Toronto, Sunnybrook Research Institute at Sunnybrook Health Sciences Centre, Ontario, ON Canada; 2 Département de Microbiologie-Infectiologie et Immunologie, Faculté de Médecine, Université Laval, Quebec City, QC Canada; 3 Ontario Veterinary College, University of Guelph, Guelph, ON Canada; 4 Department of Medical Microbiology and Immunology, University of Alberta, Edmonton, AB Canada; 5 Li Ka Shing Institute of Virology, University of Alberta, Edmonton, AB Canada; 6 Vaccine and Infectious Disease Organization, Department of Veterinary Microbiology, University of Saskatchewan, Saskatoon, SK Canada; 7 Combined Containment Level 3 Unit, Temerty Faculty of Medicine, University of Toronto, Toronto, ON Canada; 8 Department of Molecular Genetics, University of Toronto, Toronto, ON Canada; 9 Department of Laboratory Medicine and Molecular Diagnostics, Division of Microbiology, Sunnybrook Health Sciences Centre, Toronto, ON Canada; 10 Department of Laboratory Medicine and Pathobiology, University of Toronto, Ontario, Canada; 11 Department of Medicine, McMaster University, Hamilton, ON Canada; 12 Department of Medical Microbiology, University of Manitoba, Winnipeg, MB Canada; 13 Department of Pathology and Laboratory Medicine, University of Pennsylvania School of Medicine, Philadelphia, PA USA

**Keywords:** DNA vaccines, SARS-CoV-2

## Abstract

The SARS-CoV-2 pandemic is an ongoing threat to global health, and wide-scale vaccination is an efficient method to reduce morbidity and mortality. We designed and evaluated two DNA plasmid vaccines, based on the pIDV-II system, expressing the SARS-CoV-2 spike gene, with or without an immunogenic peptide, in mice, and in a Syrian hamster model of infection. Both vaccines demonstrated robust immunogenicity in BALB/c and C57BL/6 mice. Additionally, the shedding of infectious virus and the viral burden in the lungs was reduced in immunized hamsters. Moreover, high-titers of neutralizing antibodies with activity against multiple SARS-CoV-2 variants were generated in immunized animals. Vaccination also protected animals from weight loss during infection. Additionally, both vaccines were effective at reducing both pulmonary and extrapulmonary pathology in vaccinated animals. These data show the potential of a DNA vaccine for SARS-CoV-2 and suggest further investigation in large animal and human studies could be pursued.

## Introduction

SARS-CoV-2 was first reported in December 2019 in China^1,2^ and has rapidly evolved into a pandemic that has caused millions of deaths globally. Furthermore, between 10–76% of patients who recover from COVID-19 often continue to suffer from a constellation of symptoms known as post-acute COVID-19 syndrome, which will represent a future challenge to global health^3,4^. Therefore, the need to reduce the burden of COVID-19 cannot be understated. Vaccination represents an important measure that can bring the pandemic under control, and clinical trials^5,6^ have demonstrated how vaccines can prevent severe disease and limit transmission^7^. However, recent data from a US-based study comparing vaccine effectiveness has highlighted differences between the mRNA vaccines from Pfizer-BioNTech and Moderna, compared to the adenovirus-based vaccine from Johnson & Johnson. Specifically, vaccine effectiveness at preventing hospitalizations was 88–93% for the mRNA vaccines but was only 71% for the adenovirus vaccine^8^. Additionally, the emergence of SARS-CoV-2 variants has shown that vaccine efficacy can be further reduced, as outbreaks have emerged in settings of vaccinated individuals^9,10^. Moreover, clinical studies have demonstrated that neutralizing antibody titers wane over time and that individuals with co-morbidities are better protected from hospitalization and severe outcomes after receiving a booster dose^11^. These facts, combined with the need for vaccines globally indicate that the continued development of other vaccine platforms is important.

Several animal species have been shown to be susceptible to SARS-CoV-2 infection and can mimic different aspects of the disease, thereby serving as models for vaccine testing^12^. The Syrian golden hamster is an established model of SARS-CoV-2 pathogenesis and has previously been reported to support replication of SARS-CoV-2 that develops a viral load in the lungs as well as pathological lesions following intranasal challenge with the virus^13^ Additionally since viral shedding has been reported, this animal model is suitable for investigating transmission as well as antiviral countermeasures and vaccines^14–17^. Ferrets are also a useful model as they mimic a milder form of COVID-19 and can help evaluate viral transmission as well as countermeasures^18,19^. Multiple COVID-19 vaccine candidates are currently approved for use, and numerous others are under evaluation at various stages. This includes multiple DNA vaccines that have advanced into clinical trials^20^. DNA vectored vaccines have been shown to be safe and immunogenic in clinical trials for both MERS-CoV and SARS-CoV-2^21,22^. Immunization using this category of vaccines results in antigen expression and presentation by the host cell and induction of B and T-cell responses^23^. Electroporation or intradermal delivery of DNA vaccines allows for the targeting of a larger surface area of the dermis, which is likely to have large numbers of Langerhans cells and dendritic cells compared to injection into the muscle and improve antigen presentation^24,25^. Moreover, the storage requirements, relative ease of production, and ability for these vaccines to be adapted for variants make them an appealing vaccine platform. Recent findings by Seo and colleagues demonstrated protection and decreased viral shedding in DNA-vaccinated nonhuman primates as early as 4 days post-infection, suggesting a potential effect on transmission^26^. Similarly, an investigation of candidate DNA-based vaccines expressing various modified versions of the SARS-CoV-2 Spike (S) gene showed that while all vaccines reduced viral burdens to a degree, deletion of the transmembrane domain reduced immunogenicity and protection in the lower lungs^27^. These studies highlighted the need to assess modifications in the spike (S) antigen and associated effects on vaccine efficacy.

Herein, we evaluated vaccine protection provided by two candidate DNA vaccines that express the SARS-CoV-2 S protein using the hamster model of infection. Additionally, we examined whether the addition of a non-naturally occurring pentamer, 5mer4, has been shown previously to be a potent adjuvant for an influenza DNA vaccine^28^, enhancing the efficacy of our SARS-CoV-2 vaccine.

## Results

### Vaccine generation

**Fig. 1:**
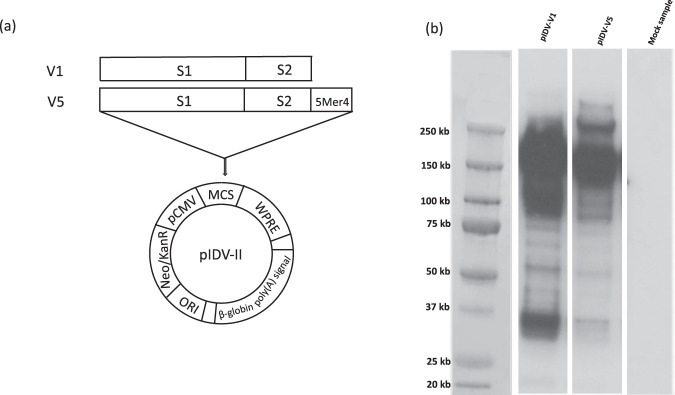
Design of plasmid vaccines. **a** Diagram showing the pIDV-II vector and antigens. Both plasmids encode codon-optimized full-length SARS-CoV-2 spike (S) open reading frame in the presence or absence of 5mer4 at 3′ end. **b** Detection of SARS-CoV-2 Spike protein in HEK 293 cells lysate by western blot. Cells were transfected with pIDV-II-SARSoCoV-Spike_v1 (pIDV-V1) and pIDV-II-SARSoCoV-Spike_v5 (pIDV-V5). The mock sample represents the 293 T cells transfected with empty vector pIDV-II; Western blot was performed using a convalescent human serum.

We generated two DNA vaccine candidates for evaluation using 92 sequences downloaded from NCBI GenBank (accessed February 24, 2020) and from which a consensus sequence was generated using MEGA X. The Consensus sequence was aligned to the S-gene from the SARS-CoV-2 isolate Wuhan-Hu-1, (GenBank accession number MN908947.3) for reference, and the codon-optimized gene was cloned into the DNA vector pIDV-II. To ensure that no spontaneous mutations in the transgene had been introduced, the sequence of the plasmid backbone and the inserted antigen was confirmed by sequencing. Two plasmids were generated, pIDV-V1 and pIDV-V5. The latter plasmid contained the 5mer4 peptide, an immunostimulatory peptide that increased the protective efficacy of an influenza vaccine (Fig. 1a)^28^. Following transfection of HEK293T cells, the lysate was harvested, and immunoblotting was performed to evaluate antigen expression, (Fig. 1b). Both vaccines expressed the SARS-CoV-2 Spike antigen (Fig. 1b).

### Immunogenicity in mice

**Fig. 2:**
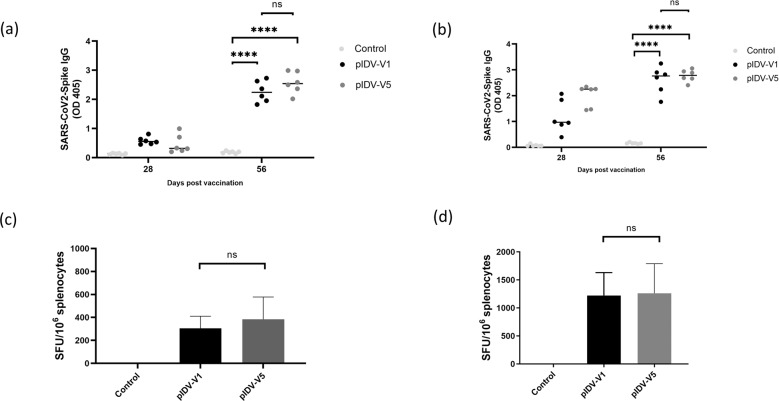
Humoral and cell-mediated responses in vaccinated mice. **a** BALB/c and **b** C57BL/6 mice were immunized with pIDV-II-SARS-CoV2-Spike-V1 (pIDV-V1), pIDV-II-SARS-CoV2-Spike-V5 (pIDV-V5), or sham vaccination with buffer only (control). Spike-specific antibodies was detected by ELISA. All mice received two doses of vaccine, one on day 0 and another on day 28 (*n* = 4 per group per timepoint). Each dose of vaccine was administered via electroporation (EP) following intramuscular injection of 50 µg/dose (ug DNA by IM + EP route). (sera dilution 1:400). **** indicates *P* value = <0.0001 as determined by one-way ANOVA. Error bars represent standard deviation. T cell response was analyzed by ELISpot 10 days after boost in **c** BALB/c and **d** C57BL/6 mice. Sham immunized mice were used as control. Splenocytes cell suspension were stimulated with SARS-CoV2 peptide pools encompassing the entire SARS-CoV2 Spike glycoprotein. No significant difference was observed for the cell-mediated response between vaccinated animals with two versions of vaccines.

To evaluate the immunogenicity of both vaccine candidates, the humoral and cellular responses generated by each vaccine were evaluated in C57BL/6 and BALB/c mice. Throughout the experiment, following vaccination, no overt adverse clinical events were observed in the animals. The humoral immune responses to the SARS-CoV-2 spike protein were investigated for both vaccines in C57BL/6 and BALB/c mice at 28- and 56-days post-vaccination. The humoral response was characterized by measuring anti-Spike antibodies in serum, and as expected mice receiving either pIDV-V1 or pIDV-V5 had higher antibodies titers than the sham-vaccinated group. The measurement of the Spike-specific IgG levels at day 56 showed a notable increase after the second vaccination, and this was observed in both mouse species (Fig. 2). Vaccination also induced a cell-mediated immune response that was evaluated by an ELISpot assay for IFN-γ producing cells, 10 days post-second vaccination. Not surprisingly, vaccinated mice produced more antigen-specific IFN-γ cells compared with unvaccinated animals, which is indicative of a robust antigen-specific cell-mediated response (Fig. 2c, d). Overall, there were no significant differences in cellular or humoral response between animals that received vaccine pIDV-V1 and vaccine pIDV-V5. Based on these data, both vaccines were carried forward for challenge studies.

### Challenge experiments and viral shedding

**Fig. 3:**
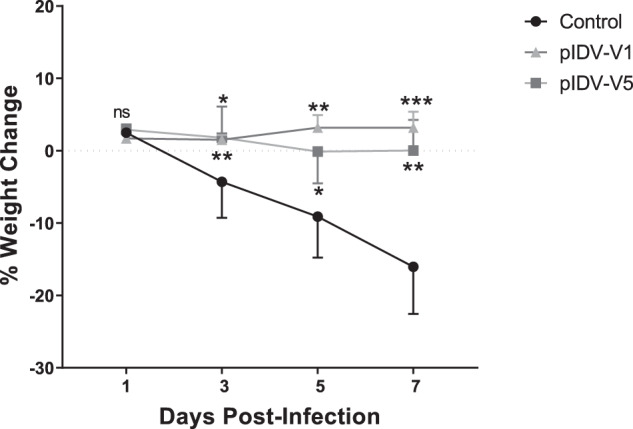
Weight loss in animals following virus challenge. Animals were weighed throughout the course of infection and weight change was compared to pre-infection weight (*n* = 8 per group). Error bars represent standard deviation. All * represent timepoints where *P* value <0.0001 as determined by two-way ANOVA.

**Fig. 4:**
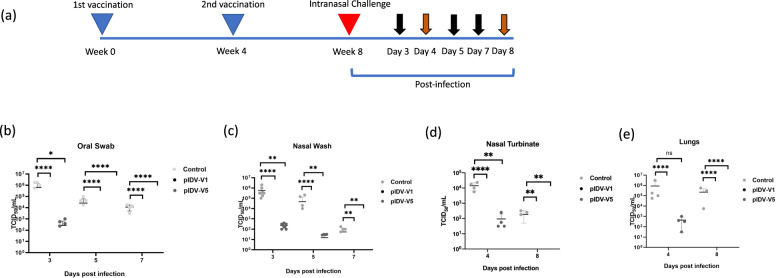
Vaccination reduces viral shedding and viral RNA load in SARS-Cov2 challenged hamsters. **a** Vaccination and challenge schedule. Sampling timepoints are shown by black arrows. Groups of animals (*n* = 4) were euthanized at 4 and 8 dpi as shown by the orange arrows. Sampling timepoints are shown by black arrows. Titers of infectious virus from various timepoints were collected and determined by TCID50 from **b** oral swabs, **c** nasal washes, **d** nasal turbinates, and **e** lungs. Error bars represent standard deviation. *P* value = 0.0001 (****) and 0.0003 (***).

Previous studies have shown that Syrian hamsters mimic multiple aspects of SARS-CoV-2 infection in humans^14,29^. We thus evaluated each vaccine for protection in Syrian hamsters. To this end, three groups of eight hamsters were vaccinated at day 0 and day 28 with either pIDV-V1, pIDV-V5, or TE buffer (sham vaccination). Four weeks after the second immunization, hamsters were challenged intranasally with a high dose of SARS-CoV-2 (8.3 × 10^5^ TCID_50_). Following infection, animals were monitored for clinical signs of disease and weight loss. Animals in the vaccinated groups showed no weight loss during the experiment. In contrast, hamsters in the sham-vaccinated group showed weight loss commencing at 3 dpi with a median loss of 8.8% by 5 dpi and maximum percentage weight loss of 16% at 7 dpi (Fig. 3). Interestingly, as shown by Tostanoski et al.^15^, infection resulted in partial mortality of animals in the unvaccinated group as one animal died on day 7. No animals succumbed to infection in either vaccinated group. No hierarchy between the two vaccine constructs could be determined based on weight loss post-challenge. Viral shedding was assessed in oral swabs and nasal washes collected post-challenge to examine potential differences between the two vaccine constructs. Both vaccines reduced the quantity of infectious viral particles compared to unvaccinated controls at 3, 5, and 7 dpi. However, no infectious virus was detected in the oral swabs or nasal washes of hamsters that had received pIDV-V1 (Fig. 4a, b). Viral shedding was also investigated in vaccinated and unvaccinated ferrets, and a similar trend was observed with no infectious virus detectable by 7 dpi in both vaccinated groups (Supplementary Fig. 1).

### Viral burden in the upper and lower respiratory tract and organs

**Fig. 5:**
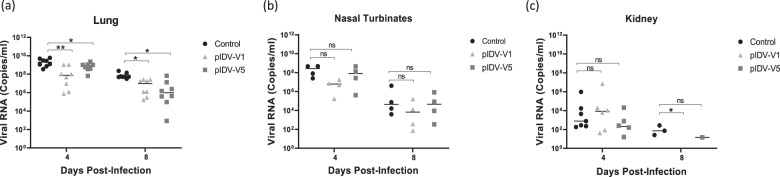
Comparison of SARS-CoV-2 viral load in different tissues. **a** Viral RNA load (mean [SD]) from hamster lung samples in control, PIDV-V1, and PIDV-V5 groups at day 4 and day 8 post-infection. Two-way- ANOVA- Dunnett’s multiple comparisons test was performed (Control vs. PIDV-V1 day 4, **p = 0.0091, Control vs. PIDV-V5 day 4 *p = 0.0457, Control vs. PIDV-V1 day 8, *p = 0.0101, Control vs. PIDV-V5 day 8 *p = 0.0119). **b** Viral load from hamster nasal turbinate samples in control, PIDV-V1, and PIDV-V5 groups at day 4 and day 8 post-infection. Two-way- ANOVA- Dunnett’s multiple comparisons test was performed (Control vs. PIDV-V1 day 4, *p* = 0.1393, Control vs. PIDV-V5 day 4 *p* = 0.5203, Control vs. PIDV-V1 day 8, *p* = 0.3616, Control vs. PIDV-V5 day 8 *p* = 0.4596). **c** Viral load from hamster Kidney samples in control, PIDV-V1, and PIDV-V5 groups at day 4 and day 8 post-infection. Two-way- ANOVA- Dunnett’s multiple comparisons test was performed (Control vs. PIDV-V1 day 4, *p* = 0.3591, Control vs. PIDV-V5 day 4 *p* = 0.3768, Control vs. PIDV-V5 day 8 *p* = 0.3432). For all panels, error bars represent the standard deviation.

While vaccination has been shown to reduce viral shedding, the quantities of infectious viral loads were also determined in the lungs and nasal turbinates following euthanasia Examination of the viral burden in lungs and nasal turbinates demonstrated significantly lower quantities of infectious virus at 4 dpi in the pIDV-V5 vaccinated group and undetectable levels of infectious virus at 8 dpi compared to the control group. In comparison, in the hamsters vaccinated with pIDV-V1, no infectious virus was detected either on 4 or 8 dpi (Fig. 4c, d). Viral RNA was detected in the lungs of both vaccinated and unvaccinated groups at both timepoints (Fig. 5). Interestingly, faster viral clearance was observed in the kidneys of vaccinated hamsters with pIDV-V1 at 8 dpi, as no viral RNA was detected at this timepoint.

### Histopathology

**Fig. 6:**
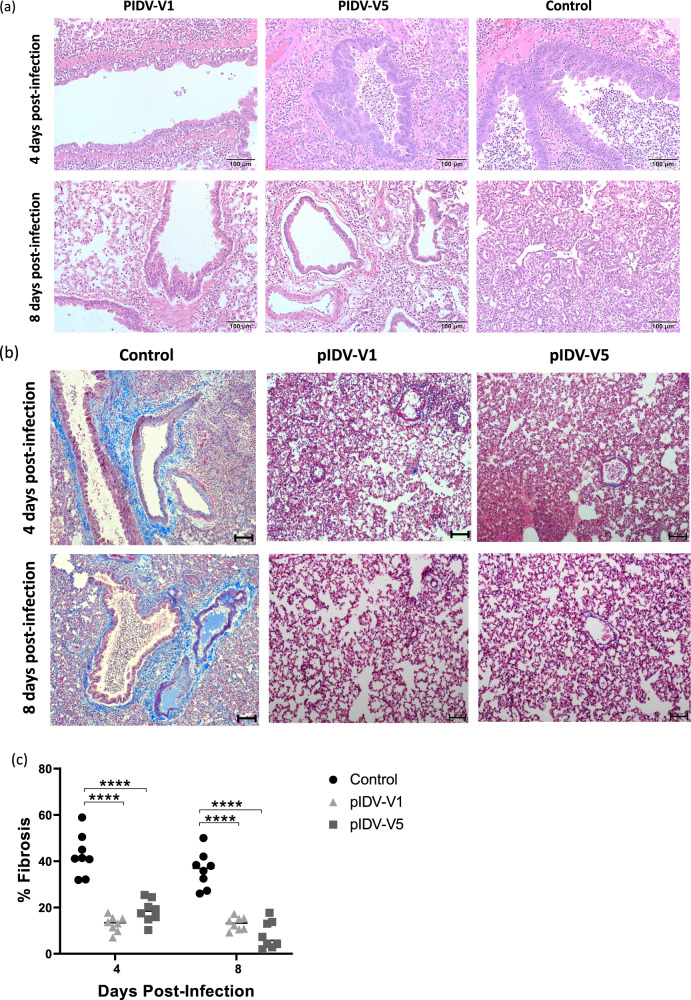
Lung pathology in vaccinated and unvaccinated hamsters. **a** Representative HE stain (10X magnification, Scale bar = 100 um) in PIDV-V1, PIDV-V5, and control groups at 4 and 8 days post-infection (*n* = 4 per group). **b** Representative Masson’s trichrome staining (10X magnification, Scale bar = 100 um). Collagen is indicated by areas stained in blue. **c** Quantification of fibrosis (collagen staining) presented as mean percentage fibrosis of total lung tissue. Two-way- ANOVA- Dunnett’s multiple comparisons test was performed (Control vs. PIDV-V1 day 4, ****p < 0.0001, Control vs. PIDV-V5 day 4 ****p < 0.0001, Control vs. PIDV-V1 day 8, ****p < 0.0001, Control vs. PIDV-V5 day 8 ****p < 0.0001). Scale bar represents 100 μm.

**Fig. 7:**
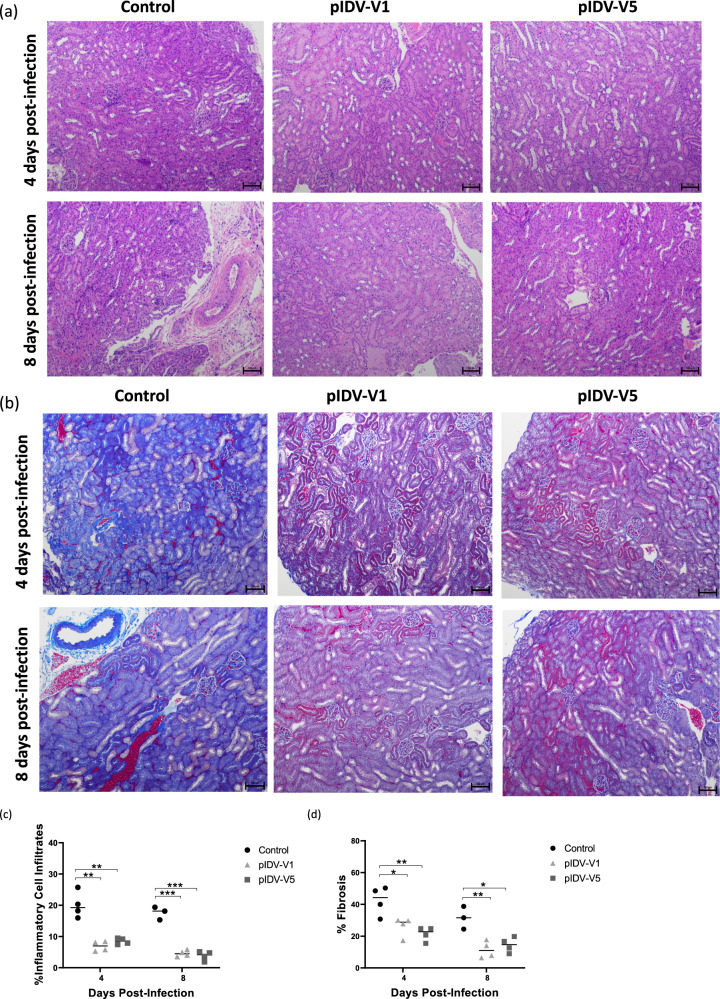
Histopathology of kidneys in vaccinated and unvaccinated hamsters. **a** Representative H&E staining (10X magnification, Scale bar = 100 um) in pIDV-V1, pIDV-V5, and control groups at 4, 8 days post-infection (*n* = 4 per group). **b** Representative Masson’s trichrome staining (10X magnification, Scale bar = 100 um) in pIDV-V1, pIDV-V5, and control groups at 4, 8 days post-infection (*n* = 4). Collagen is indicated by areas stained in blue. **c** Quantification of inflammatory cell infiltration in kidney tissues of control, pIDV-VI, and pIDV-V5 vaccinated groups. Two-way- ANOVA- Dunnett’s multiple comparisons test was performed (Control vs. pIDV-V1 day 4, **p = 0.0011, Control vs. pIDV-V5 day 4 **p = 0.0017, Control vs. pIDV-V1 day 8, ***p = 0.0001, Control vs. pIDV-V5 day 8 ****p = 0.0404). **d** Quantification of fibrosis (collagen staining) presented as mean percentage fibrosis of total kidney tissue. Two-way- ANOVA- Dunnett’s multiple comparisons test was performed (Control vs. pIDV-V1 day 4, *p = 0.0241, Control vs. pIDV-V5 day 4 **p = 0.0057, Control vs. pIDV-V1 day 8, **p = 0.0077, Control vs. pIDV-V5 day 8 *p = 0.0120). Scale bar represents 100 μm.

Following euthanasia of the animals, lungs and nasal turbinates were examined and scored to determine the extent of pulmonary disease. Pulmonary pathology was observed to a greater extent in unvaccinated animals, and disease scores were highest in this group compared to animals that received either of the vaccines (Fig. 6a). As described in Table 1, the inflammatory response and amount of lung tissue affected at day 4 were most severe in the control group (lung score, though this was only slightly higher than the hamsters vaccinated with PIDV-V5. Hamsters vaccinated with PIDV-V1 had a few lung lesions present after 4 dpi. On day 8, both vaccinated groups had reduced lung scores, with the lesions largely comprised of mildly inflammatory responses. In the control group at day 8, lung scores were also slightly reduced as compared to the day 4 controls. However, there were fewer inflammatory lesions present on day 8 along a significant regenerative response was present (Table 1). Investigation of lung fibrosis was also performed^30^. A lower percentage of fibrosis was observed in both vaccinated groups compared to the controls, with the lowest collagen deposition percentage observed in the lungs of animals that received the PIDV-V1 vaccine (Fig. 6c). In the nasal turbinates, neutrophilic intraepithelial inflammation was only observed in two control animals at 4 dpi. Overall, the pathology scores and fibrosis percentages suggested more severe disease in the unvaccinated group. Pathology in the kidneys was also evaluated, as renal tropism for SARS-CoV-2 has been described, and kidney injury is a frequent complication of infection^30,31^. Inflammation and an influx of inflammatory cells were observed to a greater degree in the control group compared to vaccinated animals at both timepoints and were suggestive of potential tubular injury or acute kidney disease (Fig. 7a, c). Furthermore, kidney fibrosis was more severe in unvaccinated than in vaccinated animals (Fig. 7b, d). Additionally, an examination of livers was performed for all groups. Similar to what has been reported by other groups^32^, greater cellular inflammation was observed in the livers of unvaccinated compared to vaccinated animals (Supplementary Fig. 2). Overall, vaccination reduced extrapulmonary pathology.

**Table 1:** Histopathology scores of hamster lungs.

	PIDV-V1 4 dpi	PIDV-V1 8 dpi	PIDV-V5 4 dpi	PIDV-V5 8 dpi	Control 4 dpi	Control 8 dpi
% Lung affected (Avg)	12.5	5	63.75	16.875	62.5	45
CTD	6	1	15	1	18	12
CVL	4	0	12	0	13	0
RIP	26	21	41	27	40	40
RR	0	0	4	3	5	16
Total	36	22	72	31	76	68

CTD: Cell/tissue damage which is comprised of bronchoepithelial necrosis (scored M1–M3), inflammatory cells/debris in bronchi (M1–M3), intraepithelial neutrophils (M1–M3) alveolar emphysema (Yes/No).

CVL**:** Circulatory/vascular lesions comprised of alveolar hemorrhage (Y/N), significant alveolar edema (Y/N), endothelial/vasculitis (M1–M3).

RIP**:** Reaction/inflammatory patterns comprised of necrosis/suppurative bronchitis (Y/N), intraalveolar macrophages (Y/N), mononuclear inflammation around airways (Y/N), neutrophilic/heterophilic inflammation (M1–M3), mesothelial reaction (M1–M3).

RR**:** Regeneration/repair, includes alveolar epithelial cell regeneration/proliferation (M1–M3) and bronchiolar epithelial cells regeneration/proliferation (M1–M3).

### Virus neutralization

**Fig. 8:**
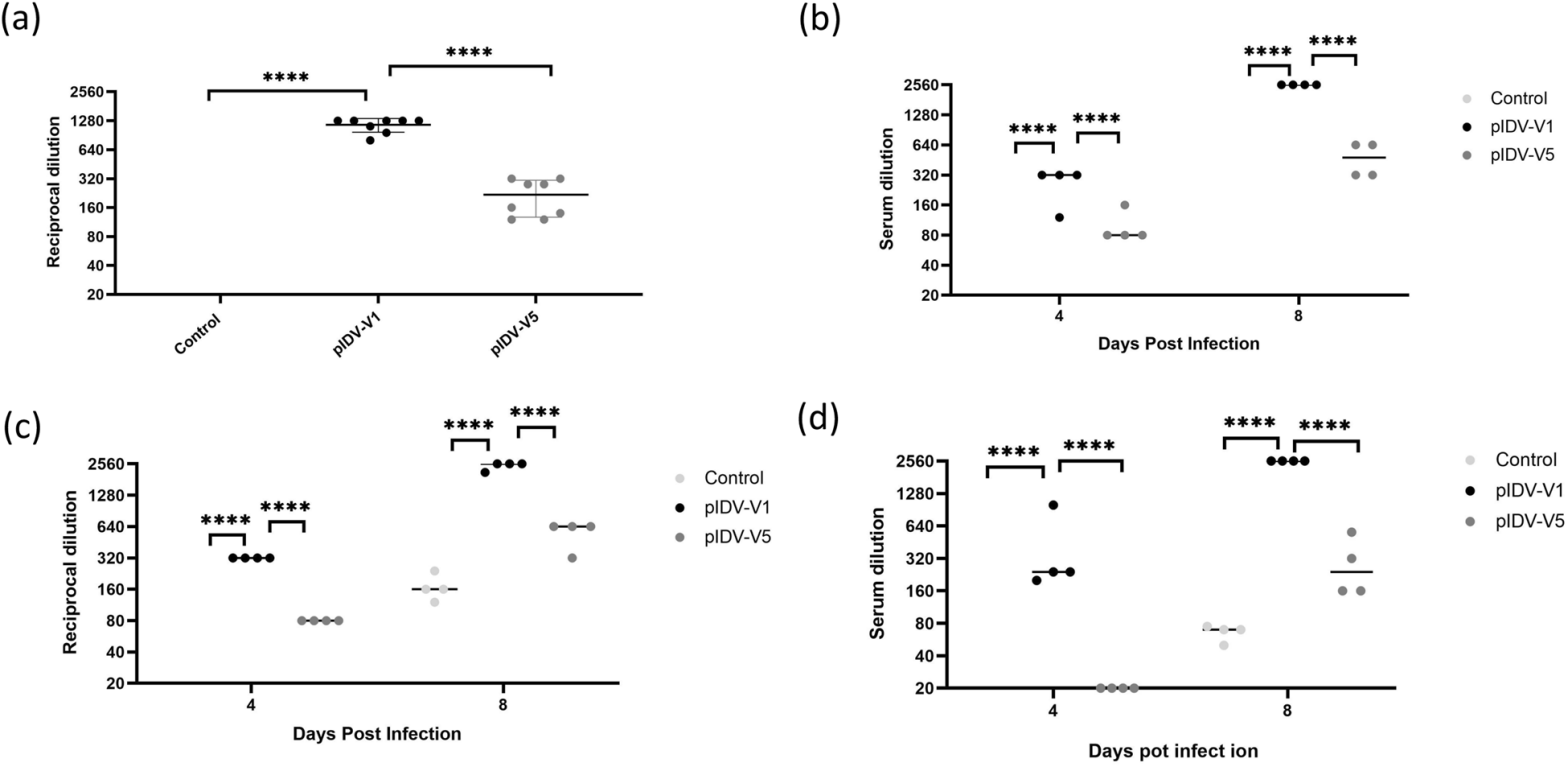
Virus neutralization. **a** Serum neutralizing titers from hamsters vaccinated with TE buffer (control), pIDV-V1, pIDV-V5 were analysed against the SB3 (B1) isolate 4 weeks after receiving the second vaccination and prior to challenge. Titers were also evaluated at 4 and 8 dpi against **b** Alpha (B.1.1.7) variant and **c** Beta (B.1.351) as well as **d** SB3 (B1). Four hamsters were used per group. Two-way ANOVA with repeated measures was used for analysis, and stars denote *p* < 0.0001.

Neutralizing antibodies are emerging as a potential correlate of protection against SARS-CoV-2^33^. To assess the ability of our vaccines to elicit neutralizing antibodies we examined titers in all groups prior to the challenge. As expected, both vaccinated groups had higher titers than the unvaccinated group. However, it was interesting to note that animals which had received pIDV-V1 had significantly higher neutralizing titers compared to animals which received pIDV-V5 (Fig. 8). Next, we evaluated viral neutralizing titers in sera from all groups of hamsters, collected at 4- and 8-days post-challenge for activity against the variants of concern. Sera from the pIDV-V1 vaccinated group was able to neutralize both the Alpha variant (B.1.1.7 lineage) and Beta variant (B1.351 lineage). Neutralizing antibodies were detected at day 4 dpi and at 8 dpi against SB3, B.1.1.7, and B1.351 in hamsters that received pIDV-V1. Interestingly, animals vaccinated with pIDV-V5 did not have detectable neutralizing at 4 dpi, and had lower levels compared to animals that received pIDV-V1 at 8 dpi (Fig. 8). Overall, pIDV-V1 vaccination resulted in higher titers of neutralizing antibodies against both variants of concern.

## Discussion

In this study, we demonstrated different levels of protection against disease caused by SARS-CoV-2 generated by a DNA vaccine expressing the Spike protein, with or without the addition of an immunostimulatory peptide. In both cases, a two-dose vaccination resulted in the production of neutralizing antibodies, lower viral loads in both upper and lower respiratory tracts, reduced viral shedding, and vaccination protected against weight loss. However, there were differences in the extent of lung and tissue pathology between the two vaccine candidates. Vaccination resulted in decreased inflammation and fibrosis in the kidneys suggesting that our vaccine candidates may limit viral dissemination to other organs. Additionally, differences in the quantity of neutralizing antibodies were noted between both vaccines in serum collected prior to challenge, as well as at 4- and 8-days post-infection. Interestingly, the version of our DNA vaccine that included the 5mer4 peptide sequence showed similar immunogenicity in mice compared to pIDV-V1which showed reduced protection in challenged hamsters. This could reflect differences in the immune response of animal models. These findings are in contrast to what has been observed with a DNA vaccine containing this peptide and expressing the HA antigen from H5N1 influenza. Patel and colleagues reported higher HA-specific titers in animals that received the vaccine with 5mer4, as well as improved survival in both mice and hamsters, suggesting 5mer4 may act as an adjuvant^28^. In the case of the SARS-CoV-2 Spike gene, the 5mer4 peptide may potentially affect the tertiary structure of S protein which could influence the antigen presentation and the immune response. Additional investigation is required to determine if any other immunostimulatory peptides identified by this group may perform better. Both of our vaccines induced antibodies capable of neutralizing SARS-CoV-2 variants and have been shown to evade immunity derived from natural infection as well as being highly transmissible and pathogenic^34,35^. Virus neutralization is a measure of antibody efficacy^33^, and a potential correlate of protection of vaccines^36^, and recent vaccination studies with a parainfluenza virus 5 vector expressing the S-antigen have demonstrated the potential of these antibodies to prevent transmission in the ferret model^37^. Our data examining viral loads in nasal washes of ferrets suggest our vaccine may also prevent transmission, although potentially later in the course of infection. Thus, future experiments will focus on whether vaccination with pIDV-V1 are sufficient to limit transmission, and if newly emerged variants such as the Delta variant are able to evade this response. Gooch et al. have demonstrated a correlation between the decrease in viral loads in the throat of NHPs, and serum neutralizing antibody titers^38^. Thus, further work is also needed to examine if mucosal antibodies are induced by our DNA vaccines.

Our vaccines have demonstrated the potential to protect against viral dissemination and multi-organ pathology as evidenced by reduced viral RNA in the kidneys of both vaccinated groups. While it has been determined that the primary cause of mortality in COVID-19 patients is acute respiratory distress syndrome (ARDS), there is increasing evidence that SARS-CoV-2 is a systemic disease that affects multiple organs, including lungs, kidney, heart, and liver^39,40^. Recent studies in hamsters by Tostanoski and colleagues demonstrated ongoing inflammation even after viral loads decreased^15^. Additionally, a recent study investigating intranasal or intramuscular delivery of a SARS-CoV-2 DNA vaccine in hamsters showed that it did not protect against lung pathology or pneumonia^16^. In contrast, both of our vaccines reduced pathology, which highlights that differences in the DNA vaccine design and route of administration are important variables and that the ability of vaccines to protect against pathology, not solely viral load, should be considered when evaluating efficacy. Our vaccine has several advantages. It is easy to generate and adapt as needed should vaccine escape variants arise and does not face the challenge of preexisting immunity that may exist with viral vectored vaccines^41^. Our dosing regimen is based on previous studies which indicated that two doses were required to generate a robust immune response^21,23^. Interestingly, we were able to demonstrate protection in our animal study using a smaller quantity of plasmid than a similar vaccine study in nonhuman primates^38^ Recent studies on individuals who received a single dose of the currently licensed mRNA COVID-19 vaccine has indicated that significant protection is conferred after one dose^42^. However, while effectiveness against the symptomatic disease was 48.7% against the Alpha variant, it decreased to 30.7% against the Delta variant^9^. Thus, future studies will investigate in animal models whether a single dose of pIDV-V1 is sufficient to protect animals against disease from circulating variants and if it can be used as a booster to supplement other vaccine platforms.

## Methods

### Vaccine candidates

Vaccine candidates were developed using the DNA plasmid platform pIDV-II that has been described previously^43^. Prior to cloning into the pIDV-II, the SARS-CoV-2 S-gene was human codon-optimized and fused to the Kozak sequence, followed by the first methionine of the antigen at the 5′amino-terminus situated right after the plasmid promoter. Western blot analyses confirmed an expression in cell lysates for all constructs. We generated vaccines expressing two versions of the SARS-CoV-2 S-gene: (V1) full length (S) and (V5) full length of (S) which subsequently was fused with short peptide 5mer4, which is a pentamer not found in the universal proteome and can enhance antigen-specific immune responses and serve as an adjuvant^28^.

### Western blotting

Briefly, 293 LTV cells transfected with pIDV-V1 and pIDV-V5 plasmids encoding SARS-CoV-2 spike glycoprotein and Spike fused with 5mer4 respectively. Cells were lysed at 48 h post-transfection by xTractor™ Buffer (Takara Bio USA, Inc.) according to the manufacturer’s instructions. Following this, lysates were centrifuged at 12,000 × *g* for 20 min at 4 °C. Next 5 µg of cleaned samples were separated in a Bis-Tris, 1.0 mm, Mini Protein Gel (Life Technologies, Canada), and transferred onto nitrocellulose membranes using the iBlot 2 Gel Transfer Device (Life Technologies, Canada). The membrane was blocked overnight at 4 °C with PBS containing 5% milk and 0.1% Tween 20 (Bio-Rad, Canada) and then blotted with a mixture of mouse Anti-SARS-CoV S Protein 154 C IgM (BEI Resources, USA), 240 C IgG2a (BEI Resources, Manassas, USA), 540 C IgG2a (BEI Resources, Manassas, USA), and 341 C IgG2a (BEI Resources, Manassas, USA) (Supplementary Fig. 1). A goat anti-mouse human peroxidase-conjugated labeled antibody (Invitrogen, Canada) was used as the secondary antibody, followed by visualization using the Clarity ECL Western Blotting Detection Substrate (Bio-Rad, Canada). The unprocessed gel is shown in Supplementary Fig. 3.

### Animals and viruses

Six-to-eight-week-old female C57BL/6 and BALB/c mice and Syrian hamsters were purchased from Charles River. Animals were housed at the BSL2 facility in Université Laval and transferred to the BSL3 facility at the University of Toronto for challenge experiments. Animal experiments described in this study were performed in compliance with the Canadian Council on Animal Care guidelines and approved by the Animal Care Ethics Committee at the Université Laval (protocol # 2016096-1), and the University of Toronto (APR-00005433-v0002-0). The virus used for animal challenge studies was passage 3 of SARS-CoV-2/SB3-TYAGNC, which was isolated from a patient that returned to Canada in March 2020^44^. For neutralization experiments, SARS-CoV2/Alberta-LKS/B1.351 or SARS-CoV2/Alberta-LKS/B.1.1.7 were isolated from residual material from a patient nasal swab and cultured initially in Vero E6 that expressing TMPRSS2 (JCRB, Japan) in DMEM containing 2% FBS, 100 IU/ml penicillin, and 100 µg/ml (Gibco, Canada), and passage 4 and 2 viral stocks, respectively, were used respectively experiments.

### Immunization and challenge studies

For immunogenicity experiments, mice were injected with 50 μg per caudal thigh of either of the plasmid DNA vaccines diluted in Endotoxin-free TE buffer (Thermo Fisher Scientific, Mississauga, Canada) or with the equivalent volume of Endotoxin-free TE buffer (sham vaccination) using intramuscular electroporation (Inovio Pharmaceutical)^38^. A total of 100 μg was administered to each animal. All mice received a boost vaccination 28 days later. For challenge experiments, groups of hamsters were vaccinated initially with either of the two vaccine candidates or with TE buffer. For vaccination 200 µg of DNA in a total volume of 200 µl endotoxin-free TE buffer (Thermo Fisher Scientific, Mississauga, Canada) was delivered into the back by intradermal (ID) delivery using an intradermal oscillating needles array injection device (Gomez et al. manuscript in revision). Briefly, a pressurized vaccine was injected into the skin by a needle array composed of hypodermic needles. The two electromagnetic coils move the needle array up and down at approximately 100 Hz. A valve controls vaccine injection and its opening is synchronized with the needle array oscillation, thereby ensuring that the vaccine is delivered when the needles are inside the skin. Animals were vaccinated, received a booster dose 28 days later, and then challenged 4 weeks after the second vaccination. Hamsters were challenged intranasally with SARS-CoV-2/SB3-TYAGNC (dose 8.3 × 10^5^ TCID_50_)^44,45^. Nasal washes and oral swabs were collected on 3, 5, and 7 days post-infection (dpi) in DMEM, while tissue samples were collected following euthanasia of animals at 4 dpi and 8 dpi, respectively. The 50% tissue culture infective dose (TCID_50_) was determined using the Reed and Muench method^46^.

### Ferret studies

Six-month-old female ferrets were purchased from Charles River and were housed at the BSL2 facility in Université Laval for vaccination. Experiments were performed in compliance with the Canadian Council on Animal Care guidelines and approved by the Animal Care Ethics Committee at the Université Laval (protocol # 2016096-1). Ferret (*n* = 8 per group) were vaccinated initially with either of the vaccine candidates or PBS. Animals were vaccinated using the intradermal method described above and received two doses 28 days apart of 1000 µg of DNA in a total volume of 1000 µl of endotoxin-free TE buffer.

Ferrets were challenged with 2 × 10^6^ TCID50/mL of SARS-CoV-2/Canada/ON/VIDO-01/Vero’76/p.2 (GISAID EPI_ISL_425177) via the intranasal route. Following the challenge, animals were monitored for weight loss, changes in body temperature, and signs of disease. Nasal washes were collected as indicated collected and infectious viral titers were determined in a manner similar to the one described above and TCID_50_ values were determined using the Spearman–Karber method^47^. All procedures were approved by the University of Saskatchewan Animal Research Ethics Board (AREB) in accordance with the guidelines of the Canadian Council on Animal Care (CCAC).

### Interferon-gamma (IFN-γ) ELISpot assay

Four mice from each group were euthanized at day 10 after boost to assess the cell-mediated response. Splenocytes from mice were assessed for SARS-CoV2 Spike antigen response via IFN-γ enzyme-linked immunospot (ELISPOT) assay, performed according to the manufacturer’s instructions (BD Biosciences, Mississauga, Canada). Briefly, cells were seeded in a Millipore plate, at 5 × 10^5^ splenocytes per well and stimulated overnight at 37 °C in 5% CO_2_ with peptide pools (JPT Innovative Peptide Solutions, Germany) containing 158 peptides derived from SARS-CoV2 and spanning the complete spike protein. The peptide pools was applied at a final concentration of 1 μg/ml. After 24 h of stimulation, plates were washed with 1x PBS containing 0.05% Tween 20, before subsequent incubation with biotinylated anti-mouse IFN-γ Ab. Then, plates were washed, streptavidin–horseradish peroxidase (HRP) was added to each well and incubated for 1 h at room temperature. The plates were washed four times and IFN-γ–secreting cells were detected using AEC Chromogen (BD biosciences, Canada). The plates were then rinsed with distilled water and dried at room temperature overnight. Results were expressed as spot forming units (SFU) per 1 × 10^6^ cells and analysis was performed using the CellProfiler software 4.1 3 (www.cellprofiler.org).

### ELISA

Blood was obtained from mice via the lateral saphenous vein 28 days after the first vaccination and again 28 days after the second vaccination. Serum was separated by centrifugation Plates were coated and incubated overnight at 4 °C with a 20 µg per plate of NR-722, a truncated and glycosylated recombinant form of the SARS-CoV-2 spike (S) external envelope glycoprotein (BEI Resources, Manassas, USA) diluted in 1X PBS. The following day, plates were washed and blocked with KPL Milk-Blocking Solution for 90 min at 37 °C. All washes were done with 1X PBS containing 0.05% Tween 20. After blocking, the plates were washed and mice sera with the dilution of 1:400 were added. Plates were incubated at 37 °C for 90 min, washed, and then incubated with anti-mouse-HRP secondary antibody at a 1:2000 dilution. Next, the plates were washed, and the substrate was added as per manufacturer instructions (KPL two-component ABTS substrate) and incubated for 25 min at 37 °C. The reaction was stopped by adding 50 µl of 1% SDS per well. Absorbance at the 450 nm wavelength was determined. Sera from naïve mice was used as an internal control on each assay group. A plate cutoff value was determined based on the average absorbance of the naive control starting dilution plus two standard deviations. Only sample dilutions whose average was above this cut-off were registered as a positive signal.

### Quantification of viral load

Quantities of infectious virus was determined by adding liquid from collected swabs and washes or homogenized tissues in DMEM to Vero E6 cells. Cells were seeded into 96-well plates and incubated overnight at 37 °C. On the following day, media was removed, and samples were added and serially diluted using tenfold dilutions into subsequent wells, Following this, the plates were incubated at 37 °C for 1 h. After incubation, the media was removed and replaced with complete DMEM, and the cells were incubated at 37 °C for 5 days. Cells were examined for cytopathic effect (CPE) at 5 dpi. TCID_50_ was defined using the Reed and Muench method^46^.

### RT-qPCR

The viral RNA loads from oral swabs, nasal washes, and homogenized tissues were extracted using the QIAamp viral RNA kit (QIAGEN, Canada) according to the manufacturer’s instructions. Detection and quantification of the SARS-CoV-2 viral RNA was performed using the Luna Universal Probe One-Step RT-qPCR kit (New England Biolabs, Canada) with the primers and probe for the E-gene, and the thermocycling conditions that have been described by Corman and colleagues^48^ on the Rotor-gene Q platform (Qiagen, Germany). For quantification, standard curves were generated using dilutions of synthetic plasmid containing a segment of the E-gene (GeneScript, USA) and interpolation was performed as has been described by Feld and colleagues^49^. The limit of quantification was determined to be 20 copies/mL.

### Virus neutralization assays

Titers of neutralizing antibodies from hamster serum was determined using the methods described by Abe and colleagues^50^. Briefly, serum specimens were diluted twofold from 1:20 to 1:2560 in DMEM supplemented with 1% penicillin and incubated with 400 TCID _50_ of the stock virus at 37 °C and 5% CO_2_ for 1 h. After incubation, this was added to 96-well plates containing confluent Vero E6 cells and were incubated at 37 °C and 5% CO_2_ for 1 h. Following this, the liquid overlay was removed and replaced with DMEM containing 2% FBS and 1% P/S. Plates were examined for CPE after 5 days and virus neutralization titers (VNT) were recorded as the reciprocal of the highest dilution of serum where the cytopathic effect (CPE) was recorded.

### Histopathology

Tissues were fixed in 10% neutral phosphate-buffered formalin, routinely processed, sectioned at 5 μm, and stained with hematoxylin and eosin (H&E) and Masson’s trichrome for histopathologic examination. Sections of nasal turbinates and lungs (left and right lobes) were examined and scored by a board-certified veterinary pathologist who was blinded to groups and days of sampling. Nasal turbinates were evaluated for the presence of intraepithelial neutrophils. Lungs were evaluated for the presence or absence of features of cell or tissue damage (necrosis of bronchiolar epithelial cells (BEC), inflammatory cells and/or cellular debris in bronchi, intraepithelial neutrophils, alveolar emphysema), circulatory changes and vascular lesions (alveolar hemorrhage, significant alveolar edema, vasculitis/vascular endothelialitis), reactive inflammatory patterns (necrosuppurative bronchitis, intraalveolar neutrophils, and macrophages, mononuclear infiltrates around airways, presence of polymorphonuclear granulocytes, perivascular mononuclear cuffs, and mesothelial reactivity), as well as regeneration and repair (alveolar epithelial hyperplasia/regeneration, BEC hyperplasia/regeneration)^51^. After the scoring was completed, the pathologist was unblinded and nasal turbinate and lung pathology scores (“Inflammation Score”) were calculated as the number of lesions present per group for each timepoint. Scores for the control group at 8 dpi were adjusted as only three control animals were available for evaluation at this timepoint.

For kidneys and liver, H&E and Masson’s Trichrome staining were also performed to assess tissue architecture and inflammation and gauge the progression of fibrosis, respectively. Images were acquired on a Nikon microscope with NIS Elements AIR 5.02.00 software under 10x objective. Non-overlapping fields of view were taken to image the entire tissue for each section. Inflammation and fibrosis were assessed by a blind observer. The region of aggregation of inflammatory cell infiltrates is delineated and represented as a percentage of the cell infiltrates area to total tissue area. The regions of collagen content stained in blue were delineated and represented as percentage fibrosis area to total tissue area. Scoring was performed by a clinical evaluator blinded to the identities of the samples

### Statistical analysis

All figures were generated using Prism version 9.0 (GraphPad Software Inc.), and statistical analysis was performed using one-way ANOVA or two-way ANOVA tests, and *p* values of <0.05 were considered significant.

### Reporting Summary

Further information on research design is available in the Nature Research Reporting Summary linked to this article.

## Supplementary information

REPORTING SUMMARY

Supplementary Figures

## Data Availability

The data that support the findings of this study are available from the corresponding author upon reasonable request.
