# Supplementary material for: Two DNA vaccines protect against severe disease and pathology due to SARS-CoV-2 in Syrian hamsters
Source: NPJ Vaccines. 2022 Apr 26;7:49. doi: 10.1038/s41541-022-00461-5 (PMC9042934; doi:10.1038/s41541-022-00461-5)
Supplement: Supplementary file 1 — REPORTING SUMMARY [file 41541_2022_461_MOESM1_ESM.pdf]

nature portfolio

Corresponding author(s): Robert Kozak

Last updated by author(s): Dec 6, 2021

Reporting Summary

Nature Portfolio wishes to improve the reproducibility of the work that we publish. This form provides structure for consistency and transparency in reporting. For further information on Nature Portfolio policies, see our Editorial Policies and the Editorial Policy Checklist.

Statistics

For all statistical analyses, confirm that the following items are present in the figure legend, table legend, main text, or Methods section.

n/a	Confirmed	
☐	☒	The exact sample size (n) for each experimental group/condition, given as a discrete number and unit of measurement
☐	☒	A statement on whether measurements were taken from distinct samples or whether the same sample was measured repeatedly
☐	☒	The statistical test(s) used AND whether they are one- or two-sided<br>*Only common tests should be described solely by name; describe more complex techniques in the Methods section.*
☐	☒	A description of all covariates tested
☒	☐	A description of any assumptions or corrections, such as tests of normality and adjustment for multiple comparisons
☐	☒	A full description of the statistical parameters including central tendency (e.g. means) or other basic estimates (e.g. regression coefficient) AND variation (e.g. standard deviation) or associated estimates of uncertainty (e.g. confidence intervals)
☐	☒	For null hypothesis testing, the test statistic (e.g. F, t, r) with confidence intervals, effect sizes, degrees of freedom and P value noted<br>*Give P values as exact values whenever suitable.*
☒	☐	For Bayesian analysis, information on the choice of priors and Markov chain Monte Carlo settings
☒	☐	For hierarchical and complex designs, identification of the appropriate level for tests and full reporting of outcomes
☒	☐	Estimates of effect sizes (e.g. Cohen's d, Pearson's r), indicating how they were calculated

Our web collection on statistics for biologists contains articles on many of the points above.

Software and code

Policy information about availability of computer code

Data collection	No software was used
Data analysis	Data analysis was performed using GraphPad Prism v9

For manuscripts utilizing custom algorithms or software that are central to the research but not yet described in published literature, software must be made available to editors and reviewers. We strongly encourage code deposition in a community repository (e.g. GitHub). See the Nature Portfolio guidelines for submitting code & software for further information.

Data

Policy information about availability of data

All manuscripts must include a data availability statement. This statement should provide the following information, where applicable:

- Accession codes, unique identifiers, or web links for publicly available datasets
- A description of any restrictions on data availability
- For clinical datasets or third party data, please ensure that the statement adheres to our policy

N/A

Field-specific reporting

Please select the one below that is the best fit for your research. If you are not sure, read the appropriate sections before making your selection.

☒ Life sciences ☐ Behavioural & social sciences ☐ Ecological, evolutionary & environmental sciences

For a reference copy of the document with all sections, see nature.com/documents/nr-reporting-summary-flat.pdf

Life sciences study design

All studies must disclose on these points even when the disclosure is negative.

Sample size	Sample size was selected based space availability in the CL3 laboratory
Data exclusions	No data were excluded
Replication	Technical replicates were included for all relevant experiments where there was sufficient sample material, in addition to biological replicates for all experiments.
Randomization	Animals were selected at random to be put into separate groups (eg. vaccinated vs. unvaccinated)
Blinding	Pathologist was blinded to sample identities when doing the evaluation and scoring

Reporting for specific materials, systems and methods

We require information from authors about some types of materials, experimental systems and methods used in many studies. Here, indicate whether each material, system or method listed is relevant to your study. If you are not sure if a list item applies to your research, read the appropriate section before selecting a response.

Materials & experimental systems

n/a	Involved in the study	
☐	☒	Antibodies
☐	☒	Eukaryotic cell lines
☒	☐	Palaeontology and archaeology
☐	☒	Animals and other organisms
☒	☐	Human research participants
☒	☐	Clinical data
☒	☐	Dual use research of concern

Methods

n/a	Involved in the study	
☒	☐	ChIP-seq
☒	☐	Flow cytometry
☒	☐	MRI-based neuroimaging

Antibodies

Antibodies used	mouse Anti-SARS-CoV S Protein 154C IgM (BEI Resources, USA), 240C IgG2a (BEI Resources, Manassas, USA), 540C IgG2a (BEI Resources, Manassas, USA) and 341C IgG2a (BEI Resources, Manassas, USA)
Validation	ATCC, on behalf of BEI Resources, hereby represents and warrants that the material provided under this certificate has been<br>subjected by the vendor to the tests and procedures specified and that the results described, along with any other data provided in this certificate, are true and accurate to the best of ATCC's knowledge

Eukaryotic cell lines

Policy information about cell lines

Cell line source(s)	ATCC
Authentication	None of the cell lines were authenticated
Mycoplasma contamination	Cell lines were not tested for mycoplasma contamination
Commonly misidentified lines (See ICLAC register)	None

Animals and other organisms

Policy information about studies involving animals; ARRIVE guidelines recommended for reporting animal research

Laboratory animals	Golden Syrian Hamster, female, 6-8 weeks old; C57BL/6 mice, female 6-8 weeks old; BALB/c mice female, 6-8 weeks old, Six month old female ferrets (Mustela furo)
Wild animals	None
Field-collected samples	None
Ethics oversight	Animal Care Ethics Committee at the Université Laval and University of Toronto. University of Saskatchewan Animal Research Ethics Board

Note that full information on the approval of the study protocol must also be provided in the manuscript.
