# Supplementary material for: Two DNA vaccines protect against severe disease and pathology due to SARS-CoV-2 in Syrian hamsters
Source: NPJ Vaccines. 2022 Apr 26;7:49. doi: 10.1038/s41541-022-00461-5 (PMC9042934; doi:10.1038/s41541-022-00461-5)
Supplement: Supplementary file 2 — Supplementary Figures [file 41541_2022_461_MOESM2_ESM.pdf]

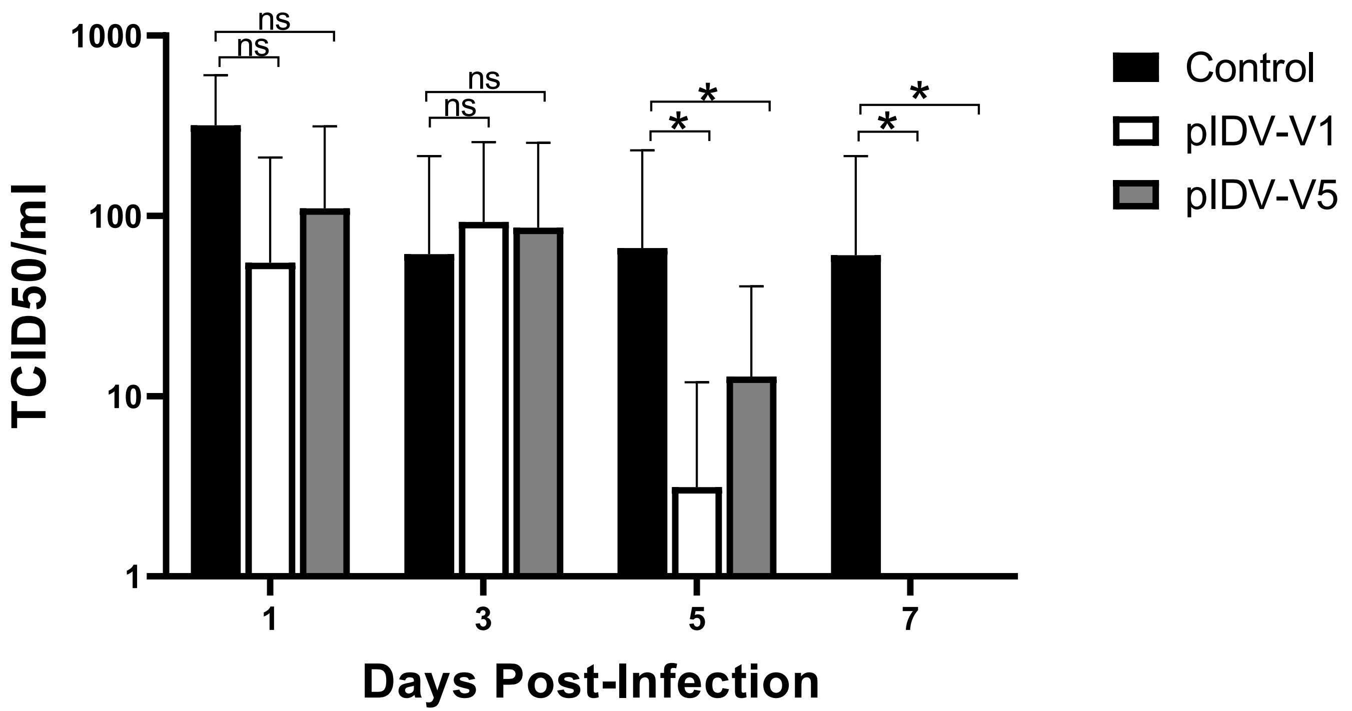


**Supplementary Figure 1. Vaccination reduces viral shedding in SARS-Cov2 challenged ferrets.** Sampling was performed on at 1, 3, 5 and 7 dpi (n=8 per group). Titers of infectious virus from nasal washes at each timepoint was determined by TCID50. Error bars represent standard deviation. **3**Two-way- ANOVA- test was performed (Control vs. pIDV-V1 day 1, p=0.1, Control vs. pIDV-V5 day 1 p= 0.117, Control vs. pIDV-V1 day 3, p= 0.7, Control vs. pIDV-V5 day 3 p= 0.76, Control vs. pIDV-V1 day 5, p=0.026, Control vs. pIDV-V5 day 5 p= 0.035, Control vs. pIDV-V1 day 7, p= 0.01, Control vs. pIDV-V5 day 7 p= 0.01).

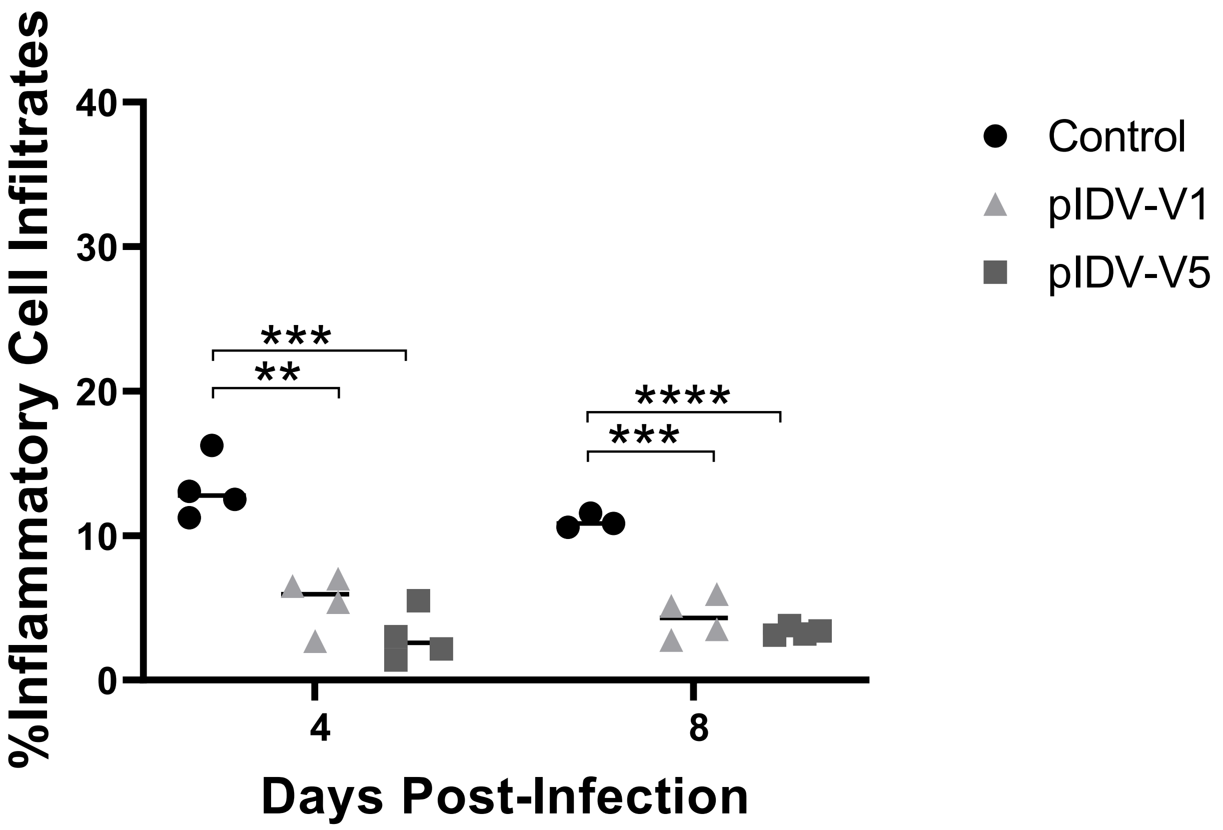


**Supplementary Figure 2. Histopathology of Hamster's Livers.** Quantification of inflammatory cell infiltration in Liver tissues of control, pIDV-VI and pIDV-V5 vaccinated groups. Quantification of cell infiltrates presented as mean percentage of inflammatory cell infiltrates in total kidney tissue + standard error of the mean. Two-way- ANOVA- Dunnett's multiple comparisons test was performed (Control vs. pIDV-V1 day 4, **p = 0.0015, Control vs. pIDV-V5 day 4 ***p = 0.0003, Control vs. pIDV-V1 day 8, ***p = 0.0007, Control vs. pIDV-V5 day 8 ****p<0.0001).

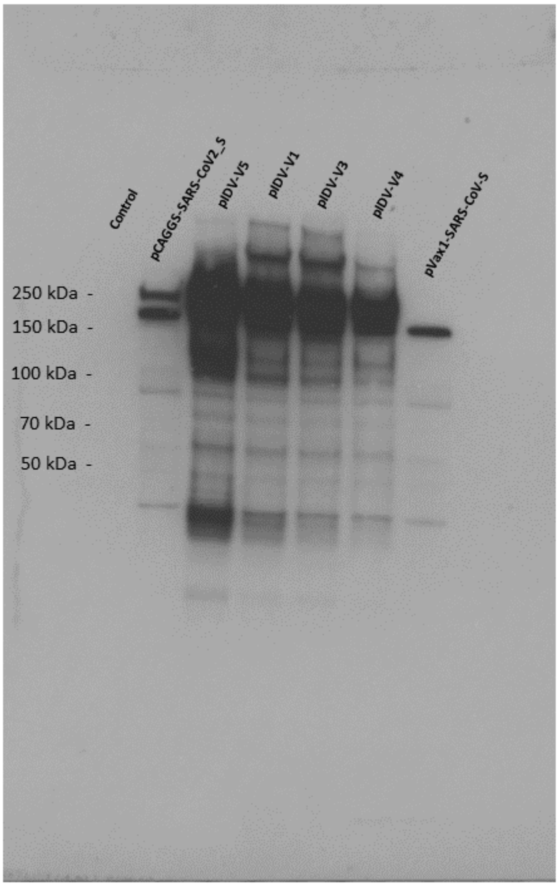


Supplementary Figure 3. Unprocessed Western blot.
